# Asymmetric Polarization in a Rough Multilayer: Towards the Discrimination of Enantiomer Pairs

**DOI:** 10.3390/nano14131109

**Published:** 2024-06-28

**Authors:** Giuseppina Simone

**Affiliations:** University of Naples ‘Federico II’, Naples, Italy; giuseppina.simone@unina.it

**Keywords:** multilayer, molecular analysis, enantioselectivity

## Abstract

Chirality plays a significant part in many vital processes, and to further our level of understanding, there is a steadily growing interest in enhancing the yield of enantioselective processes. Here, a multilayer with etched grooves is activated in a Kretschmann geometry and consists of alternating platinum Pt, silica SiO_2_, and silicon Si, as well as a silver Ag layer. Due to the production process, the groove surface exhibits a micrometric roughness, characterized by a typical vibrational mode at ω = 96 MHz. The mode is attributed to a localized acoustic vibration and has been detected as a transmitted signal. The outcomes of the inquiry include plasmonic amplification of the transmitted signal and its wavevector-less nature; in addition, it is shown that the signal is depolarized in reference to the incident beam because of the rough surface. When the Kretschmann scheme is combined with the depolarization brought on by the roughness, a built-in asymmetry results in a higher optical flux of spectrum photons in the depolarized plane than the co-polarized plane, resulting in distinct, enantioselective, and solely polarization-dependent spectral contrast. In conclusion, enantioselectivity is demonstrated for the D,L-penicillamine.

## 1. Introduction

Enantiomers of chiral compounds are widely distributed in nature; among biomolecules with chiral centers, homochirality occasionally predominates and affects the life functions [1,2,3] or confers deleterious effects over the advantages of the specular pair. Then, a high degree of selectivity is requested. Chirality encompasses geometrical considerations and appears in the optical characteristics and behavior of the systems, causing circular polarization of the light in the right or left hand, circular dichroism, or optical rotatory dispersion [4]. Due to their differing refractive indices, the enantiomers are optically active and cause a phase shift between their two orthogonal optical planes [5,6]. Yet, because they have subwavelength dimensions, chiral phenomena are weak and occasionally difficult to detect. Circular dichroism [7] and optical rotation [8], as well as chromatography [9], are the most common techniques applied for differentiating between the enantiomers. The separation occursin solution with a solvent that has been opportunely selected for not interfering with the properties of the target molecules. In turn, when an enantiomeric pair is excited in a standard Raman, the power of discrimination is minimal because the focused cone is symmetric, and then, the total focal volume, and the intensity ratio between the orthogonal polarization planes—that is, the co-polarized and the depolarized—are comparable for both enantiomers [10]. In turn, low-frequency spectroscopy (e.g., low-frequency Raman) can monitor both Stokes and anti-Stokes and enables the differentiation between signal and noise, which is useful for the identification of the enantiomers and extensive information on higher-order structure, global variations, and intermolecular interactions.

In the late 1970s, the link between Raman and surface plasmon resonance was demonstrated [11,12], and for the first time, a Kretschmann geometry was adapted for Raman spectroscopy. When coupled in a Raman configuration, the resonant excitation of the surface plasmon polaritons resulted in an angular and polarization dependence [13,14,15] as well as in a dramatic enhancement of the scattering cross-section, when the analytes were adsorbed on rough metal surfaces. The photon tunneling in the total internal reflection geometry had to match the photon and surface plasmon polariton wavevector to provide the necessary wavevector conservation. This allowed the signal to be used to analyze molecular rotation by converting the generated quanta of the polaritons to photons via a dynamical Casimir effect. Based on this principle, it seemed possible to overcome the limitation of the yield of discrimination between the paired objects [16].

In this study, a low-frequency vibrational mode that is caused by the rough metal surface has been examined. The vibration is attributed to the localized acoustic vibration of the rough features of a multilayer made up of alternating platinum (Pt), silica (SiO_2_), and silicon (Si), as well as silver (Ag). After conducting an initial exploration of surface plasmon resonance, the analysis of the transmitted signal was carried out, by exciting the system at a selected wavelength to induce the excitation of polaritons in a Kretschmann configuration. As demonstrated by the analytical and experimental findings, the multilayer has a typical mode at a frequency of ω = 96 MHz and the transmitted signal is enhanced due to plasmonic resonance, a phenomenon associated with the oscillation of electrons at the surface of a metal nanostructure [17]. The amplification is influenced by the presence of micrometric features decorating the surface, as well as by the acoustic waves propagating through the material. A complex interplay between the structural characteristics of the surface and the acoustic properties of the medium is suggested, potentially offering insights into novel signal amplification mechanisms. Both observations brought the conclusion that the transmitted signal’s nature is related to an intermediate state that is present close to the rough surface, as shown by the peak intensity difference between on-resonance and out-of-resonance measurements, and that an intermediate state is hypothesized to be stretched in free space rather than being situated at the surface due to the dependence on the refractive index of the surrounding medium. The crucial behavior of this signal is to be generated by a rough surface and be depolarized with reference to an incident beam. Indeed, the depolarization caused by the roughness, when combined with the Kretschmann arrangement, enables a built-in asymmetry to produce a higher optical flux of spectrum photons in the depolarized plane than the co-polarized plane. The prevalence of the depolarization prospects a discrete, enantioselective, and exclusively polarization-dependent spectral contrast. The specific configuration has the advantage of operating independently of the pair of enantiomers. Based on the results presented, it appears that this method can be applied to a wide range of different pairs of chiral molecules, which allows for broader potential applications and greater versatility in chemical analysis and research.

## 2. Rationale for Experiments

**Figure 1:**
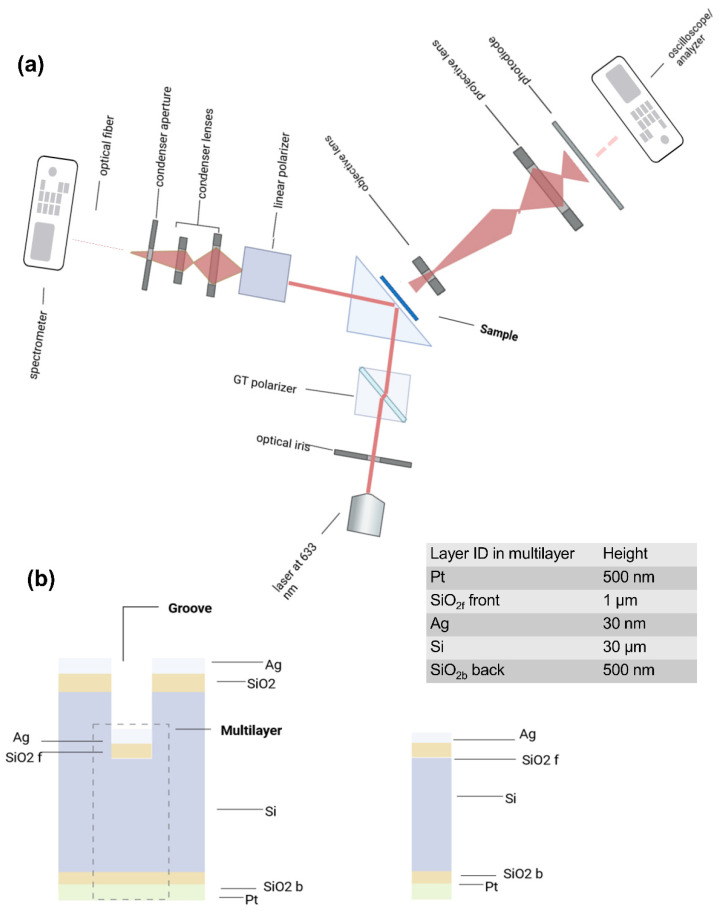
The experimental information. (**a**) The setup and working principle. (**b**) An out-of-scale schematic cross-section of groove and multilayer. The inset shows the focus on the multilayer architecture and a table listing the geometry.

The detection configuration comprises two moduli [18,19,20]: reflection for surface plasmon resonance and transmission. A detailed description of the experimental setup geometry is displayed in Figure 1a.

The reflectance detection is located at the back of the prism. It uses a double Glan-Taylor Calcite Polarizer (Thorlabs Inc., Newton, NJ, USA) to control the polarization plane of the excitation beam after it has been collimated (Thorlabs Inc. RC12FC-P01); the asymmetric focal cone is created by adjusting the excitation of a continuous beam at various incident angles. The structure is illuminated with a transverse, *p*-polarized, 633 nm beam with a power of 5 mW. The reflected signal is linearly polarized and collected by a photodiode, then analyzed by an oscilloscope. The transmission module is housed in the front part of the prism. The light is collected by a silicon photodiode (FDS 10×10 Thorlabs, 340–1100 nm, peak at 960 nm responsivity 0.62 A/W), and a ceramic disk capacitor reduces the noise [21]. The photodiode collects the signal and sends it to an oscilloscope before the transformation of the signal in the frequency domain by a spectrum analyzer (PicoTech, series 5000) is applied.

**Figure 2:**
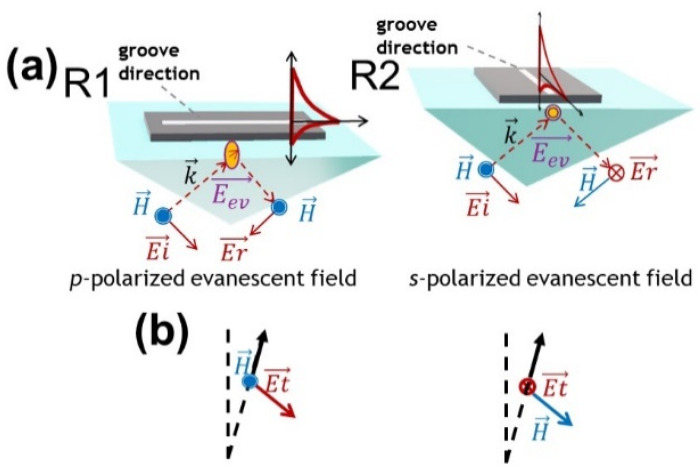
Arrangement of the sample in the Kretschmann setup. (**a**) The sample/beam arrangement. (**b**) The orientation of the vector of the transmitted electric and magnetic field. Symbols: E, H: electric and magnetic vector, k: wavevector. Subscript symbols: i: incident, r: reflected, ev: evanescent, t: transmitted.

The cross-section of the device in Figure 1b includes an optical active module, a detailed multilayer (shown in the right part of Figure 1b), and an etched groove for holding the sample, which is optically inactive. The fabrication of the sample has been widely described in [22]. Figure 2 accounts for the influence of the grooves’ orientation according to the incident vectors. By mounting the samples with the prism in accordance with the two configurations labeled as R1 and R2, and exciting the samples with a *p*-polarized beam, the electric and magnetic fields change their direction due to the orientation of the evanescent wave developed inside the groove, and can depolarize the incident and reflected beams. Because of the micrometric roughness that characterizes the surface of the grooves, a transmitted signal is expected to be measured on the front side of the prism as well [23]. The discussed results will refer to the orientation of the electromagnetic vectors relative to the two configurations R1 and R2 but also to the effect of depolarization due to the micrometric roughness. The latter factor will come into play once the transmitted signal’s characteristics have been measured and examined.

## 3. Results

### 3.1. Multilayer Plasmonic Response and Transmitted Signal

**Figure 3:**
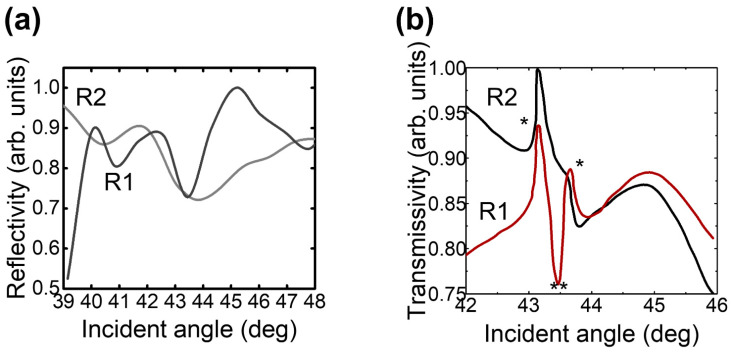
Surface plasmon resonance. (**a**) Relative reflectivity of multilayer/column system. (**b**) Transmission spectrum according to incident angle. All data were recorded in air at λ_ex_ = 633 nm. Symbol: (*) peak, (**) dip.

In the first place, the plasmonic behavior of the two configurations was investigated. According to the angular interrogation, the reflectance spectrum and relative to the R1 configuration occurred at an incidence angle of ϑ = 43.48 deg (Figure 3a). Nonetheless, the trace indicates that at least two more modes have grown [24]; in turn, the R2 configuration’s reflectance dip is slightly right shifted in relation to R1 and is broader. Because the only difference existing between the due configurations belongs to the relative orientation of the groove with the polarized exciting beam, the differences between the two signals R1 and R2 seem to be attributable to a co- and depolarization of the evanescent field. In this prospect, R1’s output and input laser are co-polarized, while R2’s reflected output is depolarized according to the exciting beam. The co-polarized R1 yields a higher signal than any other depolarized one along any other direction including the orthogonal plane R2, as confirmed by R1’s narrower linewidth.

**Figure 4:**
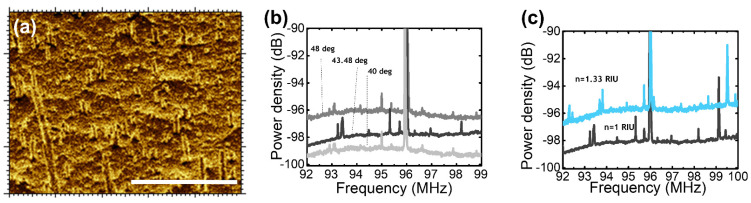
Transmission. (**a**) A high-resolution image of the surface roughness. Scale bar 100 μm. (**b**) FTT spectrum of the optomechanical hybrid system at several angles of the angle of incidence. (**c**) FTT spectrum of the optomechanical hybrid system at ϑ_res_ = 43.48 deg in an aqueous environment (ϑ_H2O,res_ = 46.5 deg).

As for the reflectance, the collection of the transmission spectra relative to R1 and R2 shows a higher signal for the co-polarized plane than in the depolarized one (Figure 3b). Moreover, R1 shows two peaks denoted with the symbol (*) separated by a dip denoted with the symbol (**), which highlights that a plasmonic-induced absorption occurs at ϑ = 43.5 deg. To explain the existence of the transmitted signal, the surface of the groove was analyzed [25]. The analysis of the surface depicted in Figure 4a yielded an average roughness of 30 ± 5 μm, with a peak-to-peak distance measured as b = 1 ± 0.3 μm. The micrometric roughness is a result of residues from the extreme anisotropy of the silicon etching process, which is defined by alternating etching and passivation phases to create the surface. An optical power of 5.0 mW was pumped into the system at an angle of incidence of 43.48 deg. The transmitted signal was collected by a photodiode and then converted into the frequency range using a spectrum analyzer. The FFT spectrum in Figure 4b shows three fundamental peaks at the resonance: a main peak centered at ω_m_ = 96 MHz and side peaks at ω ≶ ω_m_. Besides, the comparison with the off-resonance spectra shows that the intensity of the side peaks (ω ≠ ω_m_) fluctuates with the angle of incidence. The intensity reaches its maximum at the resonance angle, while some of the peaks disappear when excited out of resonance. The results emphasize the effectiveness of optical resonance in enhancing transmission.

Hence, the absence of a noticeable frequency shift between the spectra proves that this behavior is not caused by the electromagnetic effect, but rather by the inelastic scattering concentrated at the roughness. An unusual aspect of the system behavior was recorded when the refractive index of the surrounding medium was changed. The measurement was made in an aqueous environment, after recording the angle of resonance at this condition (ϑ_H2O,res_ = 46.5 deg). In Figure 4c, the impact of the refractive index of the surrounding environment is shown. When the refractive index increases (for example, when substituting air with water, where *n*_H2O_ = 1.33 RIU and *n_air_* = 1.0 RIU), the distance between the peak at ω_m_ = 96 MHz and the side peak decreases for frequencies in the range ω < ω_m_, while it increases at frequencies to the right of ω_m_. The same result was obtained when identical measurements of the transmission were repeated after various molecules diluted in water were used to fill the groove. Therefore, variations in the refractive index result in spectra having a shift relatively to the plain multilayer. Summarizing, it emerges that the typical frequency of the system is extremely low. Along with the demonstration of independence from the adsorbed molecules, the latter observation confirms that the transmitted signal is exclusively related to an acoustic vibration focused on the metallic roughness. Furthermore, it is straightforward to calculate the theoretical fundamental frequency of the vibration using the formula *f = v/d*. By incorporating the numerical experimental values, one can tentatively deduce that *f* = 100 MHz, where *v* represents the propagation speed of sound in metal (*v* = 3 × 10^3^ m/s) and *d* stands for the typical dimension of the roughness. The overlap between the experimental and analytical findings confirms the hypothesis about the nature of the transmitted signal.

Another aspect relies on the plasmonic enhancement of the transmitted signal. The peak intensity difference between on-resonance and out-of-resonance measurements highlights that the nature of the transmitted signal is related to an intermediate state lying in the proximity of the rough surface. Moreover, since there is a dependence on the refractive index of the surrounding medium, it is suggested that the intermediate state is not located at the surface but is extended in the free space in which the surface plasmon polaritons travel. According to the numerical analysis, the spatial extension of the layer or penetration depth at the air side is δ = 5.04 nm and significantly thinner than the exciting beam wavelength λ_exc_ > δ [26]. All those observations lead to the conclusion that the measured spectrum must be significant for the localized surface plasmons.

**Figure 5:**
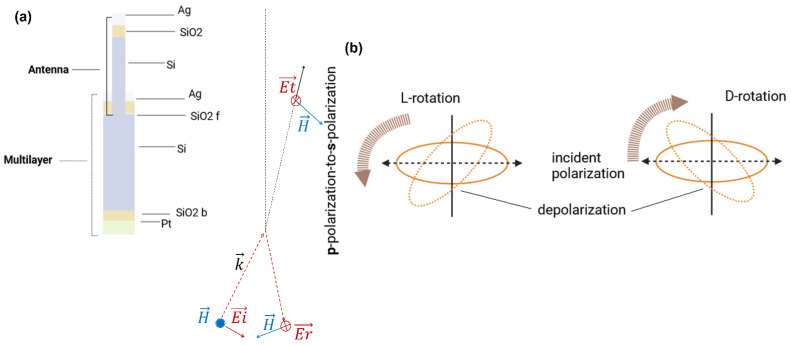
Depolarization caused by micrometric roughness. (**a**) Scheme of depolarization leads by surface roughness. (**b**) Diagram of asymmetric depolarization from combined effect of roughness excitation in Kretschmann setup.

The enhancement of the transmitted signal is not the only cause derived by the rough surface. The hypothesis is that when the system is excited by a *p*-polarized beam (Figure 5a), the light reflects and transmits in both *s*- and *p*-polarization (Figure 5b), due to depolarization caused by the surface roughness. Therefore, when considering the experimental configuration R1 for studying the transmitted signal, the s-polarized beam is collected as output as well. This has important implications for the subsequent analysis. The first implication is related to the influence that depolarization has on the shift of the transmitted modes to the low-frequency range, and its relevance to the detection of modes that are visible in these frequency ranges (such as intermolecular interactions, biomolecular bindings, and chirality). In addition, when combined with a Kretschmann configuration, the depolarization induced by the roughness [27] makes it possible for a built-in asymmetry to produce a larger optical flux of spectrum photons in the depolarized plane than the co-polarized plane, which results in a distinct, enantioselective, and solely polarization-dependent spectral contrast.

### 3.2. Discrimination of Enantiomers

**Figure 6:**
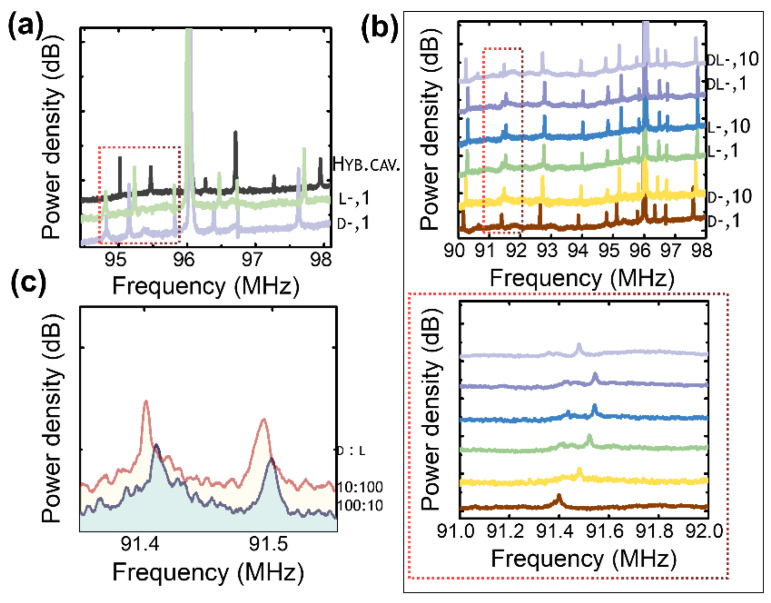
Enantioselectivity. (**a**) The emission broadband spectroscopy of a (D)-penicillamine and (L)-penicillamine in comparison with the hybrid multilayer. The highlighted area shows the frequency shift. (**b**) Transmission spectrum (D)-penicillamine and (L)-penicillamine, and racemic solution DL; 1, 10 stands for 1 mm and 10 mm. The range of frequency in the dashed rectangles is shown at the bottom of the panel. (**c**) Transmission spectrum of solutions D- (10 mm)/L (10 mm)-penicillamine, in ratios of 100:10 and 10:100.

The transmission path’s asymmetric scattering cross-section has the potential to visually highlight the distinctions between pairs of enantiomers. This property allows the system to reveal the differences between the pairs. A couple of enantiomers, D, L-penicillamine, diluted in ultra-pure water (concentration of the solution 1 mm), were injected into the clean groove before recording the transmitted pattern.The results were compared with the plain multilayer (Figure 6a). The analysis of the peaks enclosed in the dashed area display a shift when the enantiomers are sampled on the multilayer [28,29,30]. Molecular differentiation becomes achievable by observing the transition dipole moments in left (L)- and right (D)-handed enantiomers. Penicillamine’s left and right configurations have been studied thus far by testing the spectra of the two independent molecules and their racemic solutions L,D (Figure 6b). The left-handed enantiomer displays a distinctive splitting of the frequency observed at ω = 91 MHz. The splitting is visible in the magnified inset and is consistent across the two concentrations examined—1 mm and 10 mm. The behavior of the dextrose molecule is intriguing as it displays a loss of splitting, indicating a significant change in its molecular influence. However, the reappearance of the splitting in the spectrum underscores the role of the left-handed molecule in contributing to the racemic behavior, highlighting the complex interplay of molecular interactions within this system. When the L- and D-isoforms of the molecule are mixed at various ratios, the intensity of the mode at ω = 91 MHz, which distinguishes the L- molecule, increases as a function of concentration (Figure 6c). As the concentration of the L- and D-isomers in the solution increases from 10 mm to 100 mm, the ratio of the peak intensity of L to D varies between the two solutions from 0.4 to 0.8 [31,32]. The spectra suggest a difference between the D- and L-enantiomers resulting from the interaction and scattering of light and the approach can be used to distinguish between the two types of molecules.

## 4. Conclusions

In conclusion, the plasmonic resonant behavior of a multilayer with a rough surface has been comprehensively studied. The co- and depolarization of the outflow reflected beam has been observed to be influenced by the configuration of the assembly in relation to the prism and the incident beam. Co-polarized reflectance exhibited tighter linewidth and more distinct modes, which have been the result of the multilayer and the Ag film. The Ag film topology is characterized by being rough, which affects the system’s optical response. The roughness, in the first place, has caused the transmission of the laser beam that is not wavevector in nature but is greatly impacted by the on-resonance excitation. In addition, the transmitted signal is depolarized according to the incident beam because of the micrometric roughness. The overlapping of these characteristics combined in a Kretschmann geometry conferred to the system a built-in asymmetry able to produce a larger optical flux of spectrum photons in the depolarized plane than the co-polarized plane and reach an enantioselective, polarization-dependent spectral difference. Here, this behavior has allowed for identifying substantial variations in the transmitted signal of a pair of enantiomers.

## Data Availability

The raw data supporting the conclusions of this article will be made available by the authors on request.
